# MoTe_2_ Field-Effect Transistors with Low Contact Resistance through Phase Tuning by Laser Irradiation

**DOI:** 10.3390/nano11112805

**Published:** 2021-10-22

**Authors:** Geun Yeol Bae, Jinsung Kim, Junyoung Kim, Siyoung Lee, Eunho Lee

**Affiliations:** 1 Green and Sustainable Materials R&D Development, Korea Institute of Industrial Technology (KITECH), Cheonan 31056, Korea; gybae@kitech.re.kr; 2 Department of Chemical Engineering, Pohang University of Science and Technology (POSTECH), Pohang 37673, Korea; jinsungkim@postech.ac.kr (J.K.); challenge@postech.ac.kr (S.L.); 3 Inspection Business Unit (IBU), Onto Innovation, Bloomington, MN 55435, USA; narsia89@gmail.com; 4 Department of Chemical Engineering, Kumoh National Institute of Technology (KIT), Gumi 39177, Korea

**Keywords:** transition metal dichalcogenides, 2D materials, chemical vapor deposition, phase, contact resistance

## Abstract

Due to their extraordinary electrical and physical properties, two-dimensional (2D) transition metal dichalcogenides (TMDs) are considered promising for use in next-generation electrical devices. However, the application of TMD-based devices is limited because of the Schottky barrier interface resulting from the absence of dangling bonds on the TMDs’ surface. Here, we introduce a facile phase-tuning approach for forming a homogenous interface between semiconducting hexagonal (2H) and semi-metallic monoclinic (1T′) molybdenum ditelluride (MoTe_2_). The formation of ohmic contacts increases the charge carrier mobility of MoTe_2_ field-effect transistor devices to 16.1 cm^2^ V^−1^s^−1^ with high reproducibility, while maintaining a high on/off current ratio by efficiently improving charge injection at the interface. The proposed method enables a simple fabrication process, local patterning, and large-area scaling for the creation of high-performance 2D electronic devices.

## 1. Introduction

Two-dimensional (2D) materials have received much attention owing to their unique physical, chemical, and electronic properties [1,2,3,4,5]. In particular, hexagonal group-Ⅵ 2D transition metal dichalcogenides (TMDs) have received significant research interest due to their exotic bandgap opening and strong spin-orbit coupling, which shows their feasibility in various applications, such as logic transistors, memristor, piezoelectronics, spintronics, wearable/flexible devices, sensors, and valley optoelectronics, among many others [6,7,8,9,10,11]. Distorted octahedral (T) phase TMDs have recently received attention in the field because of their promising features in novel electronic devices and topological field-effect transistors (FETs) based on quantum spin Hall effects [12]. Unlike the semiconducting (2H) phase, which has a relatively high bandgap opening (≥1 eV) and high structural and environmental stability, the semi-metallic (1T′) phase with a slight band overlap near the Fermi level is often found to be metastable [12,13,14,15,16,17,18]. When this phase is used in FETs, the semiconducting layer comprises a single layer or a few layers and has a covalently bonded lattice, where all charge carriers are confined in an atomically thin channel path, resulting in excellent gate tunability and a high on/off current ratio (*I*_on_/*I*_off_). Hence, it has significant potential in the optimal scaling of transistors for single-atom devices. However, only inhomogeneous centimeter-scale 1T′ MoS_2_ has been reported [19]; this limits the size of the 1T′ TMD region for scalable applications. Thin-film tellurides, such as MoTe_2_ and WTe_2_, feature the most optimized electronic features, including superconductivity, quantum spin Hall state, and 2H, 1T, and 1T′ phases [12,20,21,22,23,24,25]. In particular, MoTe_2_ is receiving broad attention for phase-tunable memory devices owing to the small energy gap between the 2H and 1T′ phases, and strain-induced phase switching, gating, and heating have been experimentally demonstrated [26,27,28,29,30]. Despite increasing interest in tellurides, creating large-scale 1T′-phase MoTe_2_ thin films in electronic applications remains a challenge, as does precisely controlling the number of layer depositions. 

Creating an ohmic contact is another obstacle in developing high-performance ultrathin TMD devices because of the lack of surface dangling bonds and the high surface-to-volume ratio. To minimize the Schottky barrier between the semiconducting channel and metallic electrode, previous studies have focused on the contact interface, including the alignment of the work functions of Co, Sc, and Au/Ti electrodes with the conduction and valence band edges of the active layer [31,32,33,34,35]. Fermi-level pinning near the interface has been found to significantly affect device characteristics. In addition, the abundant metal electrodes near the conduction band were also found to improve device performance, owing to the effective carrier injection, which resulted in a decrease in the contact resistance [31]. For example, graphene-electrode-based FET devices show the advantages of electrical properties such as a tunable work function, high electrical conductivity, and nominal damage to the active layer. Transfer-free direct graphene synthesis on the MoS_2_ layer resulted in a barrier height of 0 meV at a high gate voltage. Consequently, a high charge carrier mobility can be obtained due to the modified contact resistance and ohmic contact of the graphene and the MoS_2_ layer [36]. The contact materials are suggested to form a highly conductive state for the active channel; this ensures a low interface resistance and facilitates carrier injection. However, doping mechanisms for nanoelectronics are typically not straightforward due to their limited doping levels, stability, and the complexity of the process [37]. Therefore, methods for obtaining two different electrical characteristics within a single element layer should be explored to fabricate high-performance electronic devices.

Here, we demonstrate a robust phase-tuning method that enables high-performance MoTe_2_ FETs with a heterophase and homojunction interface. We developed a locally patterned semi-metallic 1T′ phase on ultrathin semiconducting MoTe_2_ nanosheets by laser irradiation. Laser-irradiated 1T′ MoTe_2_ FET devices exhibited enhanced electrical performances due to the effective charge injection via the homojunction interface. Additionally, we demonstrated a contact resistance of 93 kΩ·μm. This low contact resistance resulted from the work function of the 1T′ MoTe_2_ and the 2H conduction band near the vacuum level. Furthermore, 1T′ MoTe_2_ FET devices showed better overall performance than that of the semiconducting 2H MoTe_2_ FETs. In addition, we measured the electrical properties in the atmosphere and obtained an average mobility of 16.1 cm^2^ V^−1^S^−1^, a high on/off ratio of more than 10^5^, and a subthreshold swing of 98 mV dec^−1^ from 36 MoTe_2_ FET devices. Laser-irradiation-assisted 2H–1T′ phase conversion is highly reproducible, suitable for scaling up and applying to large areas, and feasible for engineering the contact interfaces of 2D layered materials for atomically thin nanoelectronics in the near future.

## 2. Materials and Methods

The mixture of MoO_3_/NaCl (4.0 mg/2.3 mg) for MoTe_2_ synthesis was placed in a sample boat, and the target substrate (SiO_2_/Si substrate) was placed at the top of the boat. A tellurium powder in a crucible for the tellurization of MoO_3_ was placed upstream of the Ar carrier gas. After evacuation, the SiO_2_/Si substrate was heated to *T* = 750 °C at a pressure of *p* = 350 Torr.

A *p*++ Si/SiO_2_ 300 nm wafer was used as a rigid substrate and gate electrode to fabricate the MoTe_2_ FETs. Before fabricating the devices, the substrate was rinsed using ethanol, acetone, isopropyl alcohol, and deionized water. The device fabrication was completed by synthesizing a MoTe_2_ active layer according to the method described above. To pattern S/D electrodes on the MoTe_2_, the shadow mask, with various channel lengths from 30 to 200 µm, was aligned. Then, a Au (30 nm)/Ti (3 nm) electrode was deposited through the thermal evaporator. After fabrication of MoTe_2_ FETs, the laser, having the wavelength of 532 nm, was irradiated at the interface MoTe_2_ and S/D electrodes to induce phase transition. The exposure time was varied from 1 to 8 s at an ambient atmosphere.

The crystalline properties of MoTe_2_ were characterized via Raman spectroscopy (WITec Alpha 300R, Ulm, Germany), and its thickness was measured using atomic force microscopy (AFM; Bruker MultiMode 8, Camarillo, CA, USA). The chemical composition and bonding properties of MoTe_2_ were determined using X-ray Photoelectron Spectroscopy (XPS, PHI VersaProbe, Chanhassen, MN, USA). The electrical characteristics were analyzed using a Keithley 2636A source meter (Tektronix, Beaverton, OR, USA) under an ambient atmosphere.

## 3. Results and Discussion

**Figure 1:**
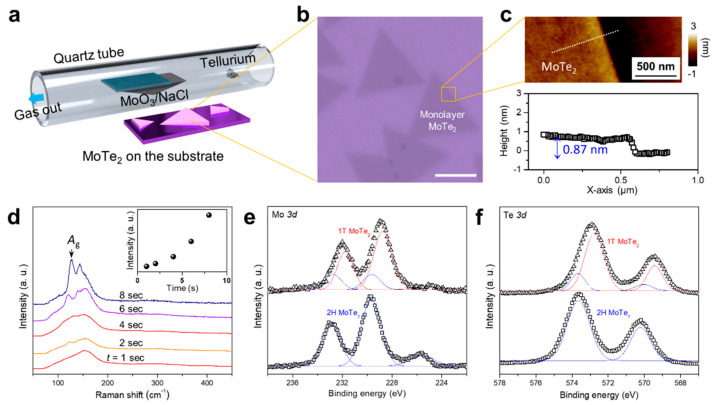
Characterization of CVD-grown MoTe_2_: (**a**) Schematic of CVD synthesis of MoTe_2_ on SiO_2_/Si substrate using salt-assisted growth; (**b**) Optical microscopy image of triangular monolayered MoTe_2_ nuclei on the SiO_2_/Si substrate. The scale bar is 2 μm; (**c**) AFM image of MoTe_2_ and the height profile corresponding to the white dashed line; the thickness of the MoTe_2_ is 0.87 nm; (**d**) Raman spectra of MoTe_2_ at various laser irradiation times. The inset shows the evolution of the Ag peak related to the 1T′ phase of MoTe_2_ with time. XPS spectra show (**e**) Mo 3*d* and (**f**) Te 3*d* peaks of the 1T′ and 2H phases of MoTe_2_.

Figure 1a shows a schematic of the synthesis of monolayered MoTe_2_ via chemical vapor deposition (CVD) on a SiO_2_/Si substrate. Figures 1b and Figure S1. show the optical images of the obtained film. The material synthesis is described in detail in the Materials and Methods section. An AFM image and a height profile (Figure 1c) show the thickness and demonstrate that the MoTe_2_ flakes on SiO_2_/Si have a height of 0.87 nm, indicating that they form a monolayer. Figure 1d shows the Raman spectra of MoTe_2_ on the SiO_2_/Si substrate after laser irradiation from 1 to 8 s. Laser irradiation causes structural phase evolution in the exposed area. All MoTe_2_ films exhibit a relatively weak peak at 235 cm^−1^ (in-plane E^1^_2g_ mode) but a strong prominent peak at 159 cm^−1^ (out-of-plane A_g_ mode) under 532 nm laser excitation [38,39,40]. The E^1^_2g_ mode signal is typically strong for 2D MoTe_2_ flakes; however, the A_g_ mode signal was higher than that of the E^1^_2g_ mode, indicating that the in-plane signal was diminished.

To obtain the critical laser irradiation time for the phase transition in the MoTe_2_ samples on the SiO_2_/Si substrate, the desired area was gradually and carefully exposed to irradiation by a laser with a power of 2.6 mW and a wavelength of 532 nm. In addition, the Raman spectra were measured after laser irradiation for each duration. The evolution trend was similar for all cases. The E^1^_2g_ peak intensity decreased gradually with increasing laser irradiation time, and when the laser irradiation time reached the critical value, two new peaks appeared at 127 and 141 cm^−1^, which correspond to the A_g_ mode of 1T′ MoTe_2_ [30]. These peaks provide direct evidence of the phase transition from the 2H phase to the 1T′ phase. The A_g_ intensity is plotted as a function of the irradiation time in the inset of Figure 1d. The critical laser irradiation time for the phase transition of MoTe_2_ from the 2H phase to the 1T′ phase was 8 s. The phase transition of MoTe_2_ might be explained by the Te vacancy formation in MoTe_2_, which results in alterations in the stoichiometry of Mo atoms; this leads to reconstruction of its atomic structure, and phase transition of the 2H phase to the 1T′ phase.

Figure 1e,f show the XPS spectra of Mo and Te, respectively. The spectra were obtained before and after laser irradiation for 8 s. As shown in Figure 1e, prominent peaks at 229.4 eV (Mo 3*d*_5/2_), 233.0 eV (Mo 3*d*_3/2_), 570.3 eV (Te 3*d*_5/2_), and 573.5 eV (Te 3*d*_3/2_) can be observed in the pristine sample, corresponding to the 2H phase. By contrast, the XPS peaks for 1T′ MoTe_2_ can be observed at 229.0 eV (Mo 3*d*_5/2_), 232.2 eV (Mo 3*d*_3/2_), 569.6 eV (Te 3*d*_5/2_), and 572.9 eV (Te 3*d*_3/2_). The binding energies of Mo and Te atoms in the irradiated area exhibit an energy shift of 0.4–0.8 eV between two different phases on MoTe_2_. This shift can be attributed to the distinct lattice symmetry of the 2H and 1T′ phases of MoTe_2_ [41]. This value is similar to previously reported energy shift values ranging from 0.4 to 0.6 eV [41,42,43]. Moreover, we observed that in 1T′ MoTe_2_, Te *3d* peaks are blue-shifted by 0.7 eV, whereas the Mo *3d* peaks are blue-shifted by 0.4 eV. It has also been previously reported that chalcogen deficiency in TMDs decreases the binding energy of the transition metal by approximately the same amount [44]. 

**Figure 2:**
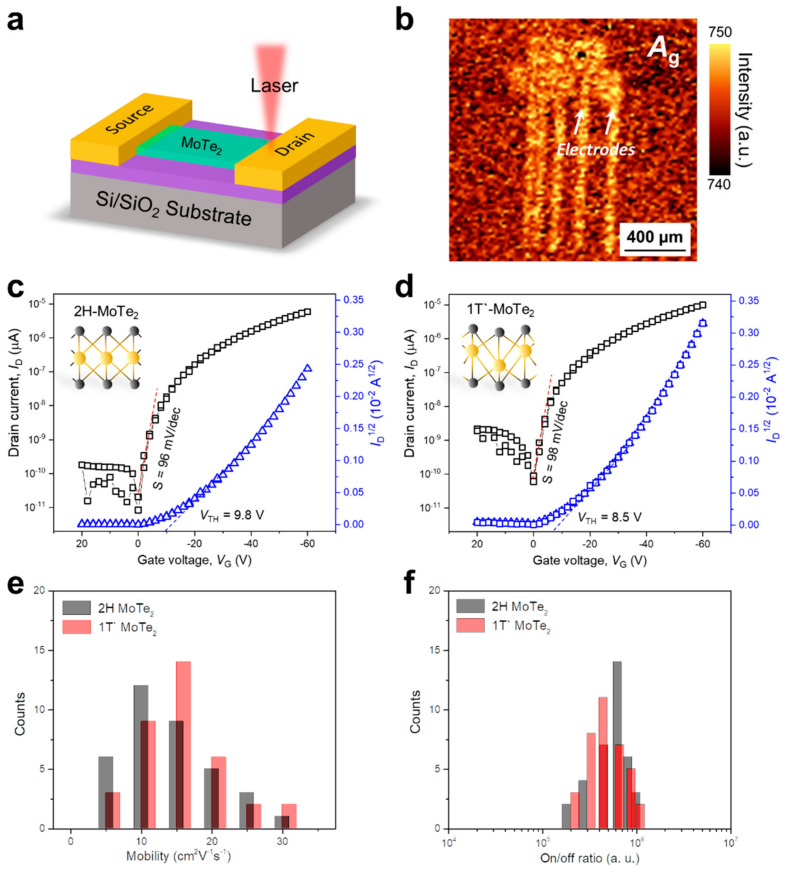
Electrical characterization of 2H and 1T′-phase of MoTe_2_ FETs: (**a**) Schematic structure of MoTe_2_ FETs on SiO_2_/Si substrate; the dimensions of the MoTe_2_ FETs are the following: channel length 20 µm and width 400 µm. (**b**) Two-dimensional A_g_ Raman mapping image of MoTe_2_ FETs on SiO_2_/Si substrate after laser irradiation. Transfer curves of (**c**) 2H MoTe_2_ and (**d**) 1T′ MoTe_2_ FETs after laser irradiation; (**e**) Statistical diagram of the measured carrier mobilities; (**f**) On/off ratio of the 36 fabricated 2H and 1T′ FETs.

To evaluate the feasibility of MoTe_2_ with a heterophase homojunction interface for electronic applications, MoTe_2_ FET devices were fabricated on a SiO_2_/Si substrate. Figure 2a,b show a schematic of the MoTe_2_ FET device structure and its A_g_ Raman mapping image after laser irradiation, respectively. The A_g_ Raman mappings show that the MoTe_2_ monolayer is not limited to deposit uniformly over the entire area but also includes the channel region, resulting in 2H to 1T′ phase-transformed MoTe_2_ devices. Overall, the laser irradiation approach efficiently enabled the fabrication of MoTe_2_ FETs by simple phase tuning. The transfer characteristics of back-gate FETs were investigated under laser irradiation. Figure 2c,d show the drain current (*I*_D_) as a function of the gate voltage (*V*_G_) of the 2H and 1T′ MoTe_2_ FETs, respectively. The measured curves show a high on/off ratio of more than 10^5^ and typical p-type transistor characteristics. From the obtained *I*-*V* curves of the MoTe_2_ FETs, we determined the field-effect mobility (*µ*) as follows:

(1) $$I_{D}=\frac{W}{2L}Cµ{\left(V_{GS}-V_{T}\right)}^{2}$$

where *I*_D_ is the drain current, *W* is the channel width of the electrode, *L* is the channel length of the electrode, *C* is the area capacitance of the SiO_2_ layer, *V*_GS_ is the gate voltage, and *V*_T_ is the threshold voltage. At the saturation regime, the average mobility values of 13.6 cm^2^ V^−1^s^−1^ for the 36 2H-phase devices and 16.1 cm^2^ V^−1^s^−1^ for the 36 1T′-phase devices were obtained, respectively; these values represent an increase of nearly 20% (Figure 2e). The high on/off ratios for the 36 2H MoTe_2_ FETs devices were well preserved after phase tuning to 1T′ by laser irradiation, as shown in Figure 2f. Moreover, 1T′ phase transformed FETs showed a high on-current level (~3.1 mA), and threshold voltage. This indicates that charge carrier movements were well preserved or improved after forming the homojunction interface. Interestingly, according to the statistical results, the on-current, as well as the off-current, slightly increased, so the carrier mobility of the 1T′ MoTe_2_ FETs was increased overall, but the average on/off ratio decreased. These results demonstrate that the heterophase homojunction interface between the active layer and electrodes enhanced the electrical performance of the MoTe_2_ FET devices and indicates the feasibility of the laser irradiation approach.

**Figure 3:**
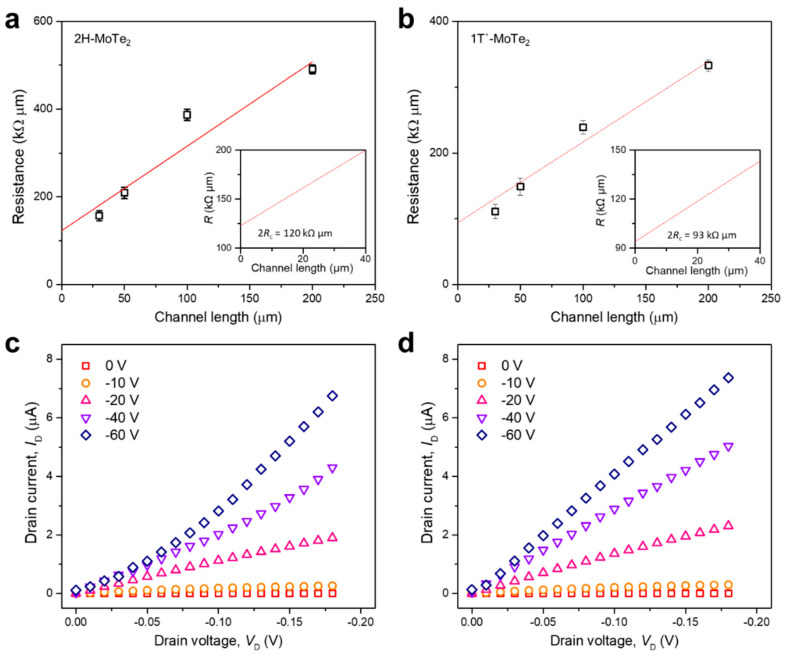
Contact resistance of the 2H-phase and 1T′-phase of MoTe_2_ FETs: (**a**) 2H MoTe_2_ and (**b**) 1T′ MoTe_2_ FETs after laser irradiation. The channel width is normalized using the resistance obtained from 2H MoTe_2_ FETs; Output curves of (**c**) 2H and (**d**) 1T′-phase MoTe_2_ FETs after laser irradiation; the FETs have gate voltages ranging from 0 to −60 V.

The contact resistances were obtained via the transfer length method (TLM) to demonstrate the reasons for the improvement in the electrical characteristics. From the TLM plots, the contact resistance values were extracted from the value for a channel length of zero. To obtain an excellent statistical agreement, at least 36 samples were measured and analyzed. Figure 3a,b show that the resistance of 1T′ MoTe_2_ decreased by 22.5%, from 120 to 93 kΩ·μm. Many studies have reported that the interface characteristics are among the main factors affecting contact resistance [45,46]. In the 1T′ MoTe_2_ device, phase tuning by laser irradiation helps reduce the contact resistance between the semiconducting layer and the electrodes. This increases the mobility and decreases the threshold voltage by reducing the contact resistance. Figure 3c shows that the 2H MoTe_2_ device exhibits nonlinear *I*_D_–*V*_DS_ characteristics, indicating Schottky behavior, whereas an ohmic-like behavior is observed in the 1T′ MoTe_2_ device performance, as shown in Figure 3d.

**Figure 4:**
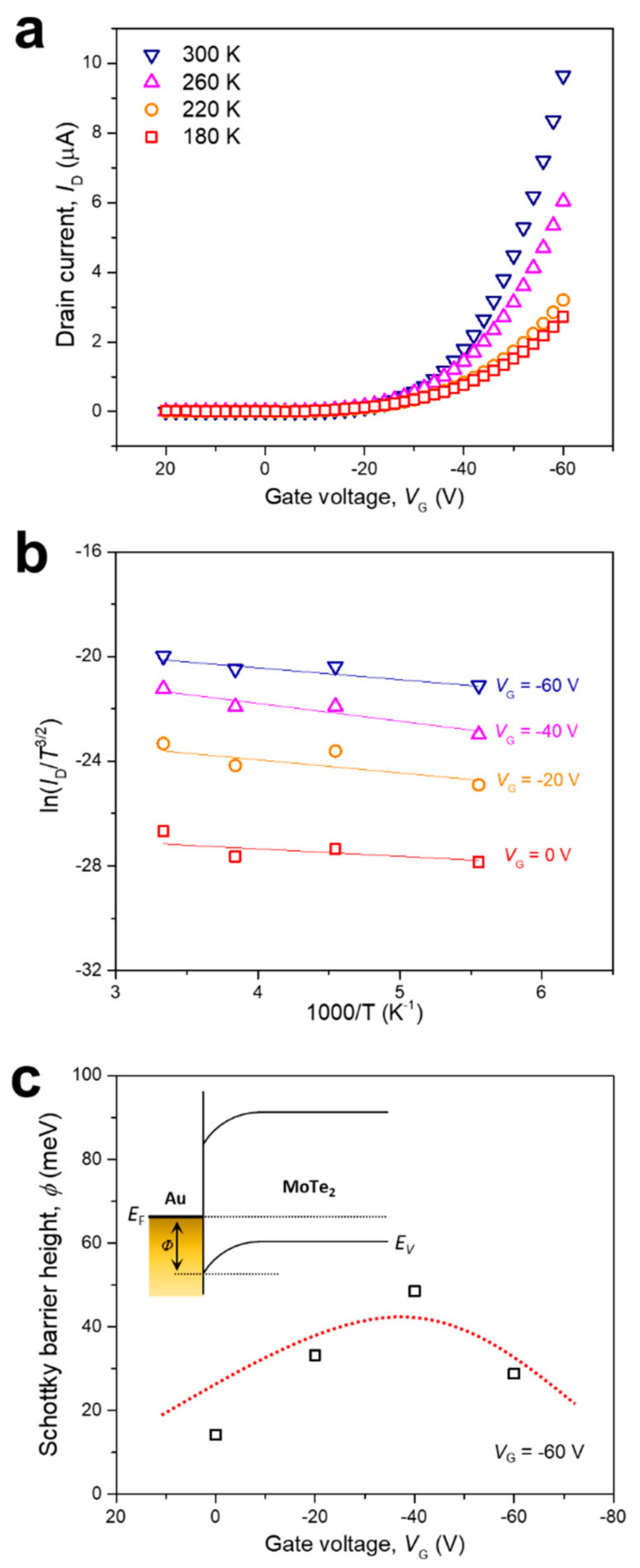
Transfer curves and Schottky barrier of 1T′ MoTe_2_ FETs at various temperatures: (**a**) Transfer curves obtained from laser-irradiated 1T′ MoTe_2_ FETs at temperatures ranging from 180 to 300 K; (**b**) Arrhenius plot of laser-irradiated 1T′ MoTe_2_ FETs with gate voltages ranging from 0 V to −60 V; (**c**) Schottky barrier height of laser-irradiated 1T′ MoTe_2_ FETs. The inset shows the schematic of the Schottky barrier of *p*-type MoTe_2_ with contact to the Au electrode.

To further quantify the ohmic contact in these devices, the barrier height at the interface between the S/D electrodes and the MoTe_2_ channel was studied [36,47]. The electrical properties were characterized at various temperatures to measure the energy levels of the metallic and semiconducting layers in the 1T′-phase MoTe_2_ devices, as shown in Figure 4.

As the temperature increases, the drain current (*I*_D_) also increases due to the hopping transport, which dominates the charge transport in MoTe_2_ [48]. The increase in the current with temperature is due to the increased charge carriers at elevated temperatures, where the thermal energy is sufficient to overcome the activation energy. The results were fitted using the following thermal emission equation to obtain an Arrhenius plot of the laser-irradiated 1T′ MoTe_2_ devices at different gate voltages:

(2) $$I_{\mathrm{DS}}=AT^{3/2}\exp\left(-\frac{q\varphi_{B}}{k_{B}T}\right)\left[\exp\left(\frac{qV_{\mathrm{DS}}}{nk_{B}T}\right)-1\right]\;\;\;\;\;$$

where *I*_DS_ is current, *A* is the Richardson’s constant, *T* is the temperature, φ_B_ is the Schottky barrier, *q* is the charge constant, *k*_B_ is the Boltzmann constant, *V*_DS_ is the drain voltage, and *n* is the nonideal factor [32,49]. When *V*_DS_ >> *k*_B_*T*, Equation (2) can be simplified as:

(3) $$\;\;\;\;\;\;\;ln\left(\frac{I_{\mathrm{DS}}}{T^{3/2}}\right)=-\frac{q\left(\varphi_{B}-\frac{V_{\mathrm{DS}}}{n}\right)}{k_{B}T}+\ln\left(A\right)$$

The Schottky energy barrier (φ_B_) can be expressed as the slope of the simplified equation in a curve of ln(*I*_DS_/*T*^3/2^) versus 1000/T. The results were plotted at different *V*_G_ values for the 1T′ MoTe_2_ FETs (Figure 4b). Finally, to obtain the nonideal factor (*n*), which is required to calculate φ_B_ for each slope, we used the following equation:

(4) $$\;\;\frac{dV_{\mathrm{DS}}}{dI_{\mathrm{DS}}}=\frac{nk_{B}T}{q}\frac{1}{I_{\mathrm{DS}}}+R_{s}\;\;\;$$

where *R*_s_ is the series resistance. We calculated n as the slope of a curve of d*V*_DS_/d*I*_DS_ versus 1/*I*_DS_ and obtained a value of 9.93 (Figure S2) [36,49]. The value of φ_B_ was calculated from these results (Figure 4c). When a zero-gate voltage was applied, the Schottky energy barrier was lower in the 1T′ MoTe_2_ device than in the 2H MoTe_2_ device, demonstrating that 1T′ MoTe_2_ forms a sharper interface between the electrode and the semiconducting layer than 2H MoTe_2_ (Figure S3). Note that the difference in transistor polarity between the 2H and 1T′ devices is explained simply by modifying the Schottky barrier height by laser irradiation. An advantage of the 1T′ device is that the S/D electrodes are side contacts rather than vertical contacts, providing better device properties [50,51]. Furthermore, this result is fundamentally different from previously reported results on chemical doping. The transistor polarity change mechanism preserves the electron and hole mobilities. It efficiently improves electron mobility with a facile approach. Moreover, 1T′-phase devices are highly independent of the metal of the S/D electrodes. We designed 1T′ MoTe_2_ devices with the same electrodes and achieved a yield of nearly 100% with consistent performance. Additionally, an ohmic-like contact was achieved at the interface between the active layer and electrodes, which improved the charge carrier injection without barriers. Although the 1T′ phase is metastable, it has been demonstrated that stability can be maintained under environmental conditions after facile laser irradiation.

## 4. Conclusions

In summary, we developed a facile method for phase tuning from 2H to 1T′ MoTe_2_ by laser irradiation. This method produces a 1T′ MoTe_2_ monolayer that facilitates charge injection at the interface and thus improves the electrical characteristics of the MoTe_2_ FETs. Moreover, we showed that phase tunability could help effectively reduce the contact resistance at the interface between the S/D electrodes and the MoTe_2_ channel, thus providing high carrier mobility of the FETs. Additionally, we demonstrated that the 1T′ phase of MoTe_2_ is a heterophase homojunction electrode with a low contact resistance value of 93 kΩ·μm at zero gate bias. The low contact resistance results in a high on-current of 3.1 mA^1/2^, and a high charge carrier mobility of 16.1 cm^2^ V^−1^s^−1^. Our work demonstrates that phase engineering is a practical approach for improving the performance of MoTe_2_ devices.

## Data Availability

Not applicable.

## Supplementary Materials

The following are available online at https://www.mdpi.com/article/10.3390/nano11112805/s1, Figure S1: Optical image of the CVD-grown MoTe_2_ on the SiO_2_/Si substrate, Figure S2: I-V curve of MoTe_2_ FETs, Figure S3: Schottky barrier height of MoTe_2_ FETs. Table S1. Comparison the device performances.
