# Supplementary material for: MoTe2 Field-Effect Transistors with Low Contact Resistance through Phase Tuning by Laser Irradiation
Source: Nanomaterials (Basel). 2021 Oct 22;11(11):2805. doi: 10.3390/nano11112805 (PMC8620056; doi:10.3390/nano11112805)
Supplement: Supplementary file 1 [file nanomaterials-11-02805-s001.zip › nanomaterials-1405986-supplementary.pdf]

Supplementary Materials

$MoTe_2$ Field-Effect Transistors with Low Contact Resistance through Phase Tuning by Laser Irradiation

Geun Yeol Bae [1,†], Jinsung Kim [2,†], Junyoung Kim [3,†], Siyoung Lee [2] and Eunho Lee [4,*]

1 Green and Sustainable Materials R&D Development, Korea Institute of Industrial Technology (KITECH), Cheonan 31056, Korea; gybae@kitech.re.kr
2 Department of Chemical Engineering, Pohang University of Science and Technology (POSTECH), Pohang 37673, Korea; jinsungkim@postech.ac.kr (J.K.); challenge@postech.ac.kr (S.L.)
3 Inspection Business Unit (IBU), Onto Innovation, Bloomington, MN 55435, USA; narsia89@gmail.com
4 Department of Chemical Engineering, Kumoh National Institute of Technology (KIT), Gumi 39177, Korea
* Correspondence: leeeh@kumoh.ac.kr
† Contributed equally.

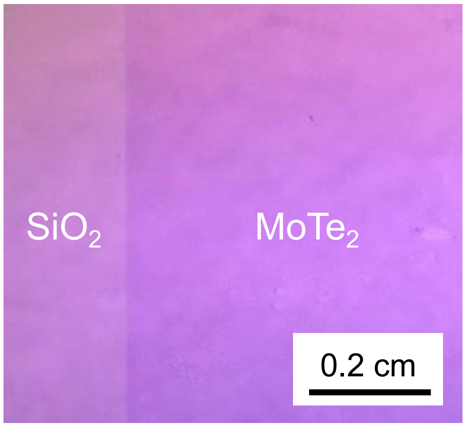

Figure S1. Optical image of the CVD-grown $MoTe_2$ on the SiO_2/Si substrate.

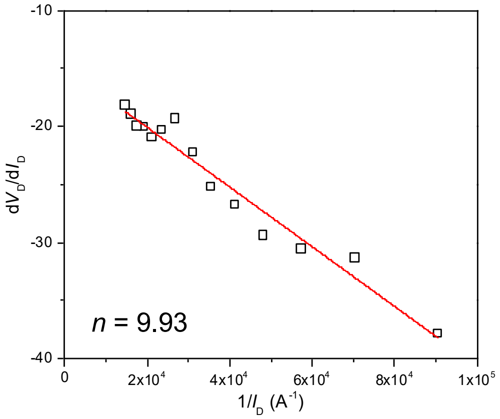

Figure S2. Plot of dV_D/dI_D as a function of $1/I_D$ of $MoTe_2$ FETs at gate voltage (V_G) of -60 V.

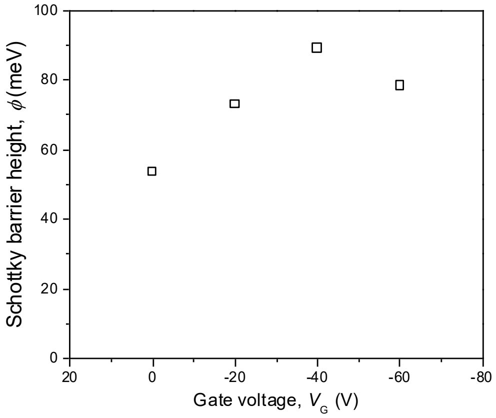

Figure S3. Schottky barrier height of $MoTe_2$ FETs without laser irradiation.

Table S1. Comparison of the field-effect transistors performances.

	Method	Mobility ($cm^2/V^{-1}s^{-1}$)	On current (mA)	on/off ratio (a. u.)
Our work	Laser irradiation	16.1	3.1	$>10^5$
J. Hwang et al. [S1]	Sputtering	8.2	$3.1x10^{-3}$	$>10^4$
J. Huang et al. [S2]	Thermal annealing	~10	$7.3x10^{-4}$	$>10^5$

References

S1. J. Huang, H. Hsu, D. Wang, W. Lin, C. Cheng, Y. Lee, T. Hou, Polymorphism Control of Layered $MoTe_2$ through Two-Dimensional Solid-Phase Crystallization, Scientific Reports, 9 (2019) 8810.

S2. J. Huang, K. Deng, P. Liu, C. Wu, C. Chou, W. Chang, Y. Lee, T. Hou, Large-Area 2D Layered $MoTe_2$ by Physical Vapor Deposition and Solid-Phase Crystallization in a Tellurium-Free Atmosphere, Advanced Materials Interfaces, 4 (2017) 1700157.
